# Advanced Nanomaterials, Printing Processes, and Applications for Flexible Hybrid Electronics

**DOI:** 10.3390/ma13163587

**Published:** 2020-08-13

**Authors:** Sehyun Park, Hojoong Kim, Jong-Hoon Kim, Woon-Hong Yeo

**Affiliations:** 1 School of Engineering and Computer Science, Washington State University, Vancouver, WA 98686, USA; sehyun.park@wsu.edu (S.P.); jh.kim@wsu.edu (J.-H.K.); 2 George W. Woodruff School of Mechanical Engineering, Institute for Electronics and Nanotechnology, Georgia Institute of Technology, Atlanta, GA 30332, USA; hkim3023@gatech.edu; 3 Wallace H. Coulter Department of Biomedical Engineering, Parker H. Petit Institute for Bioengineering and Biosciences, Neural Engineering Center, Institute for Materials, Institute for Robotics and Intelligent Machines, Georgia Institute of Technology, Atlanta, GA 30332, USA

**Keywords:** functional nanomaterials, printing of nanomaterials, flexible hybrid electronics (FHE), wearable systems, implantable devices

## Abstract

Recent advances in nanomaterial preparation and printing technologies provide unique opportunities to develop flexible hybrid electronics (FHE) for various healthcare applications. Unlike the costly, multi-step, and error-prone cleanroom-based nano-microfabrication, the printing of nanomaterials offers advantages, including cost-effectiveness, high-throughput, reliability, and scalability. Here, this review summarizes the most up-to-date nanomaterials, methods of nanomaterial printing, and system integrations to fabricate advanced FHE in wearable and implantable applications. Detailed strategies to enhance the resolution, uniformity, flexibility, and durability of nanomaterial printing are summarized. We discuss the sensitivity, functionality, and performance of recently reported printed electronics with application areas in wearable sensors, prosthetics, and health monitoring implantable systems. Collectively, the main contribution of this paper is in the summary of the essential requirements of material properties, mechanisms for printed sensors, and electronics.

## 1. Introduction

Recent advances in functional nanomaterials and printed electronics have attracted enormous interest, due to the advantages of low-cost, scalability, high-throughput, rapid processing, and hybrid integration of both inorganic and organic materials, compared to the conventional fabrication methods [1,2,3,4,5,6]. Conventional microfabricated electronics in cleanroom facilities require numerous materials and expensive sets of material processing equipment, which include subtractive manufacturing lines with toxic chemicals [7]. Moreover, these devices using semiconductor-manufacturing methods are rigid and bulky, which causes limitations in emerging applications for wearable and implantable biomedical devices. Thus, it is necessary to explore a new set of material preparation, processing, and manufacturing of electronic devices with cost-effectiveness [8,9,10] and simple processing techniques [11].

One of the emerging methods includes nanomaterial printing. Printing is an additive manufacturing process in which electronic devices can be fabricated by layer-by-layer material deposition, without using additional lithography, etching, and material evaporation [12]. This mechanism enables a roll-to-roll printing technique to manufacture industrial-scale, large-volume, electronic devices with minimal requirements of equipment and a little chemical waste [11,13]. Additionally, hybrid integration of metallic materials with various types of soft and biocompatible polymers expands the application areas of printed electronics from the electronic market to health monitoring, disease diagnosis, and therapeutics. In addition, the global market of printed electronics is expected to reach USD 26.6 billion by 2022 from USD 14.0 billion in 2017 at a compound annual growth rate of 13.6% [14].

Mechanical compliance with high bendability and stretchability of such electronics provides not only comfortable wear to users but also tissue-compatible, long-term monitoring of target signals from the human body. Recent enhancements in printing resolution, pattern uniformity, and sintering processes allow fabricating high-performance electronic components, similar to the ones from conventional cleanroom fabrication. The most popular use of printed electronics is in wearable healthcare and human-machine interfaces. Thus, some of the recent review articles summarize the novelty, functionality, fabrication strategies of the printed wearable electronics, including printing techniques [11], printable materials [4,15], sensor fabrication [3,16,17,18], and their applications [2]. These works, however, have focused on the limited scope of materials, processing, manufacturing, or applications.

**Figure 1:**
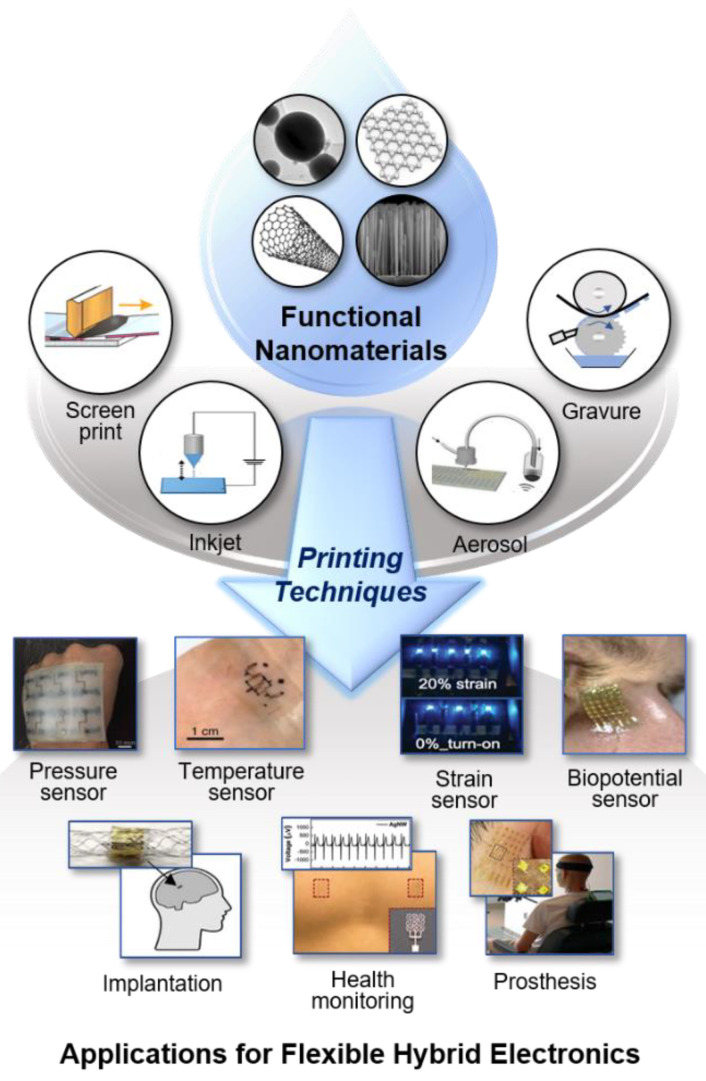
Overview of nanomaterial printing for the development of flexible hybrid electronics. Functional Nanomaterials: Nanoparticles. Reprint is in accordance with the Creative Commons Attribution 4.0 International License [19]. Graphene. Carbon nanotube. Reproduced with permission from reference [20], Copyright 2011, RSC. Nanowires. Reproduced with permission from reference [21], Copyright 2017, RSC. Printing Techniques: Screen-printing. Reprinted with permission from reference [22], Copyright 2006, American Scientific Publishers. Inkjet printing. Aerosol printing. Reprint is in accordance with the Creative Commons Attribution 4.0 International License [23]. Gravure printing. Applications for Flexible Hybrid Electronics: Pressure sensor. Reproduced with permission from reference [24], Copyright 2019, John Wiley and Sons. Temperature sensor. Reproduced with permission from reference [25], Copyright 2018, Springer Nature. Strain sensor. Reproduced with permission from reference [26], Copyright 2014, John Wiley and Sons. Biopotential sensor. Reprint is in accordance with the Creative Commons Attribution 4.0 International License [27]. Implantation. Reproduced with permission from reference [23], Copyright 2019, John Wiley and Sons. Health monitoring. Reproduced with permission from reference [28], Copyright 2018, RCS. Prosthesis. Reproduced with permission from reference [29], Copyright 2019, Springer Nature.

Here, this article delivers an all-encompassing, comprehensive review that includes nanomaterial synthesis, ink formation, various printing mechanisms, sensor characteristics, and examples of FHE in healthcare and human-machine interfaces (Figure 1). We summarize an extensive list of nanomaterial properties for the design of specific sensors and electronics, strategies for enhanced device performance (resolution, uniformity, flexibility, stretchability, and durability), and advanced sensors (temperature, strain, pressure, electrochemical, and electrophysiological). Finally, we discuss future orientations of next-generation printing methods and flexible hybrid electronics.

## 2. Printable Nanomaterials

Nanomaterials have dimensional components within 1 μm for at least one dimension [30]. These materials have shown many interesting properties to enable high-performance printed electronics. This section reviews the key properties of various nanomaterials to achieve the ideal performance of designed electronics. For example, the design of sensors and electronics has to consider the following parameters: low resistivity for electrodes and interconnections, high hole and electron mobility for semiconductors, and high transmittance for displays [6]. Optical and electrical properties that are dependent on size and shape need to be considered as well [31]. Some examples of novel synthesis of nanomaterials will be discussed, which encompass the control of size and length of nanomaterials, and their hybrid complementary hetero-materials to transcend the intrinsic disadvantages of using pure nanomaterials.

### 2.1. Metal Nanoparticles (NPs)

For printed electronics, numerous metallic NPs have been developed, including Cu [32,33,34], Au [31,35,36], Pd [37,38], Ni [26], and Ag [39,40]. Among them, Ag is one of the widely used metals due to the excellent electrical conductivity and low affinity for oxygen. Au has outstanding conductivity, stability, and biocompatibility [41]. Cu has relatively high conductivity with low cost, but the high affinity for oxygen hinders wider applications [13,40]. Table 1 summarizes the properties of representative conductive inks for printed electronics.

**Figure 2:**
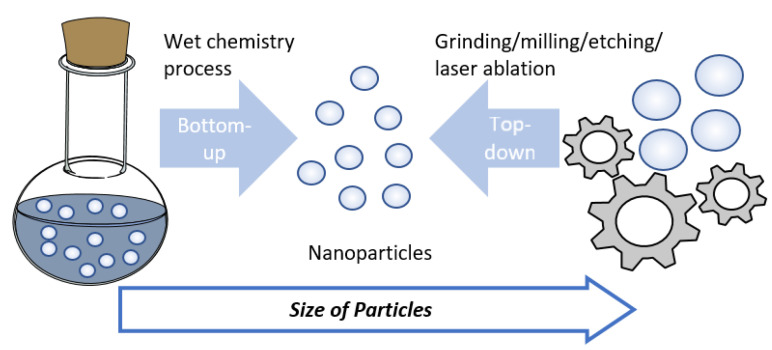
Nanoparticle preparation via the top-down method, including physical treatments such as grinding, ball milling, and laser ablation.

**Figure 3:**
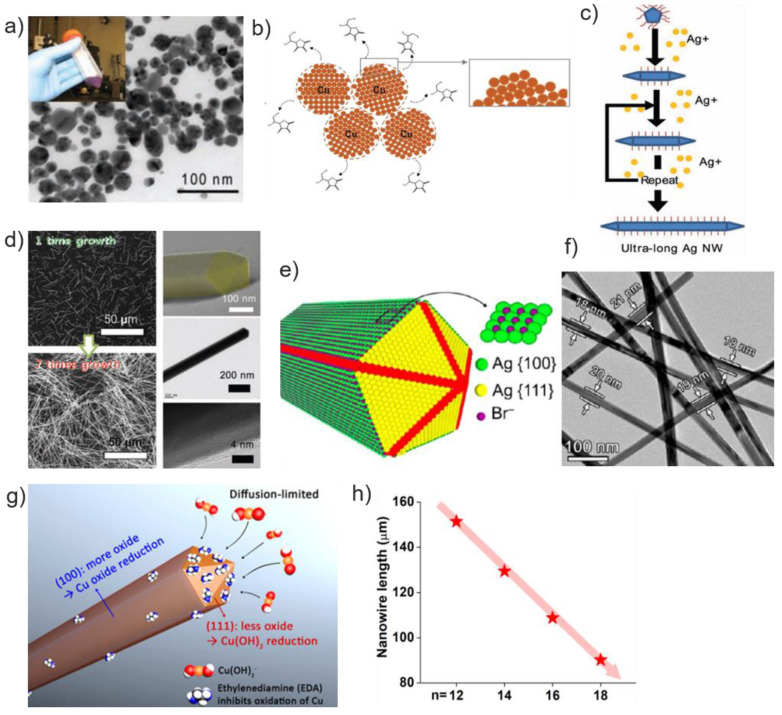
(**a**) TEM image of synthesized AgNPs and ink. Reprinted with permission from reference [45], Copyright 2009, AAAS; (**b**) illustration of synthesized CuNP reduced and stabilized in ascorbic acid. Reprinted with permission from reference [32], Copyright 2019, ACS; (**c**) schematic of the synthesis process of very long AgNW by the successive multistep growth (SMG) method. (**d**) SEM images of AgNW by the SMG method after one- and seven-times growth. SEM and TEM image of single AgNW after a seven times SMG process. Reprinted with permission from reference [50], Copyright 2012, John Wiley and Sons; (**e**) schematics of the effects of Br− ions hamstring AgNW growth of {100} direction. Reprinted with permission from reference [51], Copyright 2016 ACS; (**f**) TEM image of AgNWs after 35min synthesis. Reprinted with permission from reference [52], Copyright 2017 ACS; (**g**) schematic of CuNW growth {111} direction. (**h**) nanowire (NW) length as a function of the length of alkylamine. Reprinted with permission from reference [53], Copyright 2018 ACS.

For the preparation of printable ink, two approaches are mostly employed for the preparation of metal NPs—‘Top-down’ and ‘Bottom-up’. As shown in Figure 2, the top-down method includes physical treatments such as grinding, ball-milling, and laser ablation. This breaks down bulk metal to nanoscale particles, followed by dispersed in the proper medium [6]. The main challenge in top-down approaches is non-uniform size distribution with high production costs. On the other hand, the bottom-up method is based on the wet chemical process in which the pre-metallic precursors are decomposed by reducing agents or heating in the presence of liquid medium to make NPs [42,43]. By manipulating experimental parameters such as solvent, temperature, and concentration of precursors, the particle size distribution and morphology can be controlled [7]. For example, Zhang et al. reported the synthesis of monodisperse AgNPs for inkjet printing. By using adipoyl hydrazide and dextrose as reductants, low resistivity of 9.18 × 10^−8^–8.76 × 10^−8^ Ω m was achieved with thermal treatment at 160 °C for 30 min [44]. Using polyacrylic acid as the capping agent and diethanolamine as the reducing agent, AgNPs that have a mean particle size of 20 ± 5 nm were produced [45] (Figure 3a). Yang et al. studied the effect of the different shapes of AgNPs in printing conductive tracks. By comparing the microstructure of tracks, the result shows that conductive ink filled with a mixture of nanorods and NPs was more favorable to form a random 3D interconnected conduction network with interesting electrical characteristics [46].

In the case of metallic nanomaterial inks, the stabilization process is critical to prevent agglomeration. For stabilization of NPs, organic surfactants or polymers are coated onto the surface of the particles [6]. The sintering process is also often required to remove these additives after the printing process to increase electrical conductivity. However, the substrate of devices can be significantly damaged since this process is usually achieved at high temperatures. Kwon et al. studied the synthesis of CuNPs without theses organic additives. L-ascorbic acid, which is well known as vitamin-C, played a key role as not only reducing and capping agents but also dispersant without the need of additional polymeric stabilizers (Figure 3b). Moreover, an intermediate product, Cu_2_O NPs, are thermo-dynamically stable but optically reactive to UV region light. This property enables the photonic sintering at room temperature, which steers clear of conventional thermal sintering [32].

### 2.2. Metal Nanowires (NWs)

Metal NWs have attracted interests as next-generation materials for wearable heaters [28], solar cells [54], and transparent conductive electrodes (TCE) [55]. Among them, AgNWs and CuNWs are promising candidates to replace conventional indium tin oxide (ITO) TCEs, which are too rigid and expensive to be widely applied in wearable electronics [50]. Electrodes must have high optical transmittance, low sheet resistance, and high flexibility for wearable applications. Despite the intrinsically high thermal and electrical performance, optical transmittance is a bottleneck. The aspect ratio, which is the ratio of length to the diameter of NW, is the key parameter to enhance the overall performance of NWs [56]. This section will discuss the synthesis of metal NWs, which are devoted to improving the aspect ratio.

#### 2.2.1. AgNWs

Various approaches of AgNWs synthesis have been reported, including the polyol method, UV irradiation method, and template method [2,57,58]. Among them, the polyol method is a well-established for generating AgNWs in control of size and shape. Materials, such as ethylene glycol, 1,2-propylene glycol, or 1,5-pentanediol, play the key role as both solvent and reducing agent. The Ag precursor, which is usually AgNO_3_, is injected into a polyol at an elevated temperature for the growth and nucleation of nanostructure resulted from the reduction of Ag^+^ ion [59]. After this initial stage of the reduction process, Ag atoms form clusters [60]. These large clusters induce forming multi-twinned seeds, resulting in the formation of wire [61]. There are two ways to increase the aspect ratio: increase the length and decrease the diameter. To increase the length of AgNWs, Lee et al. developed a successive multistep growth (SMG) method to grow AgNWs over 500 μm using successive AgNO_3_ reduction in ethylene glycol solution with PVP (Figure 3c,d). Decreasing the diameter of AgNWs is also an interesting option to enhance the aspect ratio. To achieve this, Silva et al. used Br^−^ ions and PVP with a high molecular weight to synthesize AgNWs [51]. AgBr and PVP of a molecular weight of 1,300,000 g/mol were effective to restrain the NWs from the lateral growth of (100) facets and induce the formation of penta-twinned decahedral seeds, which manipulate the formation of AgNWs of (111) facet (Figure 3e). Finally, AgNWs were produced with diameters below 20 nm and aspect ratios over 1000 (Figure 3f).

#### 2.2.2. CuNWs

CuNWs are attractive materials for commercialization, due to excellent electrical conductivity and low-cost manufacturing compared to Ag and Au. Various approaches for synthesizing CuNW have been reported: chemical vapor deposition (CVD), electrochemical deposition in templates, and solution-phase reduction synthesis. Among them, the solution-phase synthesis is widely used [62]. This method requires low reaction temperature and can be performed at atmospheric pressure. The selective reduction of Cu precursors at the end of the NWs makes CuNWs grow up to tens of micrometers long. Ethylenediamine (EDA), Alkylamines are usually employed for the synthesis. EDA is a facet-selective promoter of Cu(111) growth, rather than a capping agent, which hinders the lateral growth of (100) facet [52]. At the same time, electrons from the oxidation of hydrazine (N_2_H_4_) reduce tetrahydroxocuprate (II) and dihydroxocuprate (I) complexes to make metallic Cu (Figure 3g) [52,53]. Another method uses alkylamines to control the length of CuNW by selecting different chain lengths of alkylamines. Kim et al. studied how alkylamine with different chain length plays a role in CuNWs growth. They suggested that longer alkylamine chains form a stronger passivation layer on the (111) facets at the tip of the NWs, while shorter alkylamines increase the rate of Cu reduction, producing higher yields of NWs (Figure 3h). However, Cu can easily be degraded in the presence of oxygen, which is the main limitation of CuNWs. To prevent oxidation, covering the Cu core structure with a protective shell layer (highly conductive graphene or Au) has been proposed [63]. Niu et al. reported ultrathin Cu-Au core-shell NWs with epitaxial structure [64]. The epitaxial growth of conformal, uniform, and ultrathin layers (1–2 nm) of Au shell on the surface of Cu achieves durability under a harsh condition (700 h at 80 °C).

#### 2.2.3. Post-Printing Treatment

**Figure 4:**
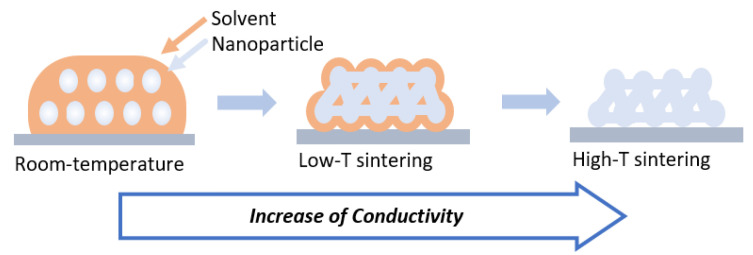
Nanoparticle preparation via the conventional thermal sintering process at high temperature.

Post-printing treatment is usually required to enhance electrical performance after printing. Metallic NPs ink includes organic additives such as a binder, dispersant, solvent. After the printing process, these organic additives typically increase the resistance of the conductive printed pattern by hindering the percolation networks of particles. The conventional thermal sintering process (Figure 4) addresses this issue. The sintering process at high temperature removes organic additives from the pattern, while inducing neck formation between adjacent NPs [6]. However, most of the flexible and stretchable substrates for FHE have a relatively low glass transition temperature, which limits the sintering temperature and time for enhancing electrical performance. To address this issue, various approaches have been reported for room temperature processing, such as electrical sintering [65], plasma sintering [66], photonic sintering [67], microwave sintering [68], and EGaIn-assisted sintering [69].

### 2.3. Carbon Nanomaterials

Carbon nanomaterials have excellent stability in a harsh environment, such as high pressure and temperature. Additionally, they are very attractive for printing electrodes for sensing due to their excellent electrical, optical, and mechanical properties and biocompatibility [18]. With these properties, carbon nanomaterials have been studied for many application areas: printed TFT [70], electrophysiological sensor [71], and micro-supercapacitor [72]. However, the main disadvantage of carbon nanomaterials is low dispersibility in many kinds of solvents. For example, the concentration of graphene in organic solvents for inkjet printing is <0.01 wt% without polymeric additives, which require multiple printing processes to stack layers for better conductivity [73,74]. This section will discuss the synthesis and dispersion of carbon nanomaterials for printing.

#### 2.3.1. Graphene

**Figure 5:**
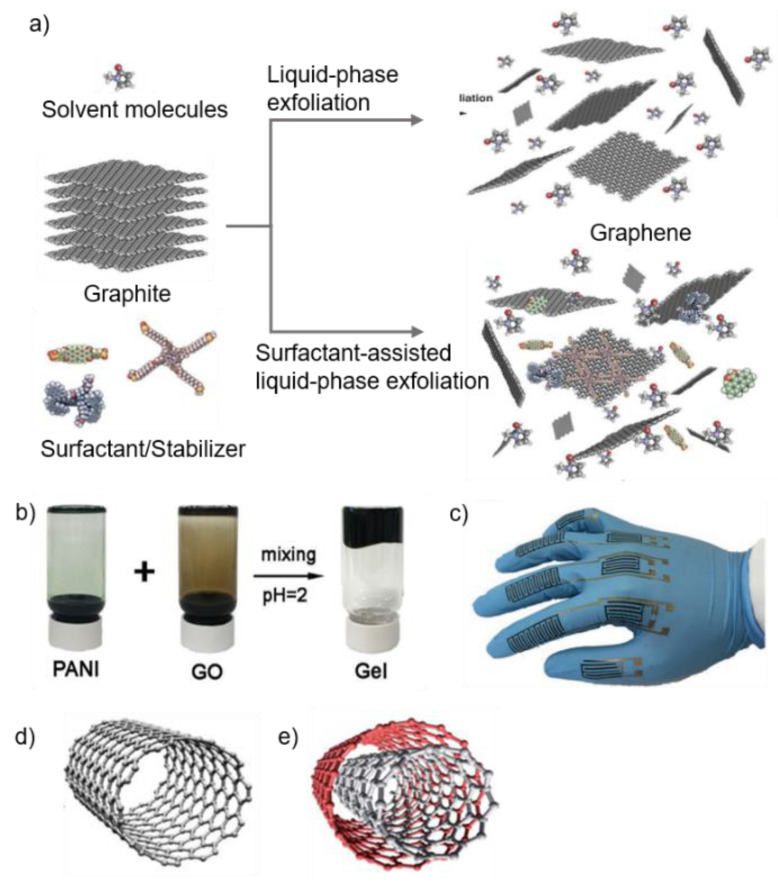
(**a**) Schematic of liquid-phase exfoliation processes of graphite with and without surfactants. Reproduced with permission from reference [75], Copyright 2014, RSC; (**b**) preparation of graphene oxide (GO)/PANI ink. (**c**) image of integrated devices printed by GO/PANI ink. Reprinted with permission from reference [81], Copyright 2019, John Wiley and Sons; (**d**) schematic of single-walled carbon nanotube (SWCNT) and (**e**) basic form of multi-walled CNT (MWCNT). Reprinted with permission from reference [20], Copyright 2011, RSC.

Since the discovery of graphene in 2004 [74], many methods have been studied to synthesize graphene. Among these, sonication assisted liquid-phase exfoliation (LPE) has been well studied to obtain high-quality graphene with low-cost and simplicity (Figure 5a). LPE processes generally include three steps: dispersion of graphite, exfoliation, and purification. The surfactant can be added to promote the exfoliation of graphite into graphene when surfactant molecules have high energy of adsorption on the basal plane of graphene [75]. The surfactant is generally small organic molecules, such as pyrene derivatives [76], perylene-based bolaamphiphiles [77], and sodium cholate [78]. Additionally, surfactants can stabilize graphene in the solvents, where the ζ potential of the surfactant-coated graphene nanosheets controls the dispersed concentration [79].

To address the dispersibility issue of printable inks, a solvent exchange method was studied. First, graphene is exfoliated from graphite flakes in the presence of DMF, and then DMF is exchanged by terpineol, where the large difference of two solvents enables the exchange by distilling them. Finally, the concentration of graphene dispersion is around 1 mg mL^−1^ [73]. Microfluidization of graphite can synthesize graphene at a high yield. Microfluidization enables almost 100% of graphite to be exfoliated to graphene while preventing them from agglomeration [80].

Graphene oxide (GO), which can be obtained by the oxidation of graphite, contains hydroxyl, epoxy, carbonyl, and carboxylic functional groups, enabling it to disperse easily in solvents without additives. Xiong et al. reported flexible, wearable micro-supercapacitor spray printing of GO and polyaniline nanofibers [81]. Easy control of the colloidal property of GO/PANI (polyaniline) gels by adjusting pH allows them to be printed with spray printing, which requires a high shear rate (Figure 5b). The micro-supercapacitor printed with GO/PANI gels shows high areal capacitance. In addition to capacitance, the printed sheets have conformal contacts on the non-planar substrate like gloves (Figure 5c). However, for conductive applications such as electrodes, GO printed patterns need post-reduction treatments such as chemical reduction or thermal treatment. These treatments often cause defects and poor electrical performance, which makes GO less attractive, compared to pristine graphene [82].

#### 2.3.2. Carbon Nanotubes (CNTs)

CNTs typically include two types: single-walled CNTs (SWCNTs) (Figure 5d) and multi-walled CNTs (MWCNTs) (Figure 5e). Due to their high conductivity and flexibility, CNTs are widely used in sensing materials and composites to improve mechanical and electrical properties. For example, soft elastomers such as PDMS (polydimethylsiloxane) have low conductivity. PDMS composites with randomly oriented CNTs can be altered to highly conductive material [18]. In another example, the printed strain sensor fabricated with AgNPs/MWCNTs nanocomposites was reported. The printed patterns show high mechanical stability (maximum strain limit of 74%) and sensing ability (GF of 58.7) [83]. Individual CNTs have great electrical conductivity. However, in most cases, CNT-based devices show much higher resistivity because defects or impurities form during the synthesis [7,84]. Moreover, their large aspect ratio causes van der Waals force to stick together, forming large clusters that can clog the printer nozzle.

### 2.4. Semiconductors

**Figure 6:**
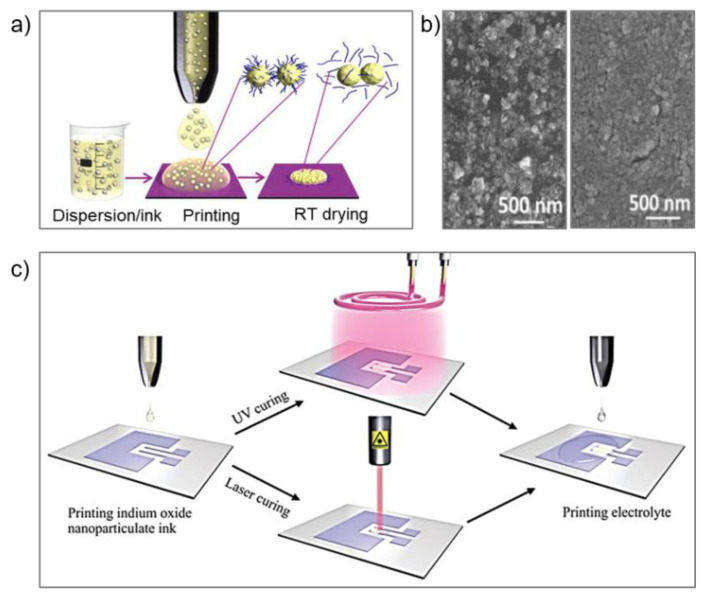
(**a**) Schematic representation of the chemically controlled destabilization and flocculation process of the printed nano ink droplets. The NaCl loaded semiconducting oxide nano inks show spontaneous stabilizer removal from the nanoparticle surface during the ink drying process. (**b**) SEM images of the printed surface topography with In_2_O_3_ nano ink and In_2_O_3_/NaCl nano ink. Reproduced with permission from reference [85], Copyright 2015, ACS; (**c**) schematic of the preparation steps of the UV-photonic cured indium oxide FETs (field-effect transistors). The process steps include printing of the indium oxide nanoparticulate ink on the FET electrodes, followed by photonic curing (UV-vis or UV-laser) of the printed nanoparticulate layers and lastly printing of the gate insulator (composite solid polymer electrolyte) to complete the fabrication process. Reproduced with permission from reference [86] Copyright 2017, John Wiley and Sons.

A sputtering process is widely used to fabricate conventional semiconductors. In recent years, solution-processing techniques have attracted interest because of low-costs, high throughput, low-temperature, and scalability, which can be realized by various printing methods. However, these solution techniques need a post-printing process to improve electron mobility, as well as conductivity. However, this high-temperature treatment is not compatible with the flexible substrate. Baby et al. investigated room-temperature processing for the semiconductor nanoparticle n-type In_2_O_3_ and p-type Cu_2_O by chemically controlling destabilization and flocculation of the NPs during the ink drying step (Figure 6a) [85]. The destabilizer NaCl successfully removes the organic stabilizer from the printed surface. This method enables the printing of a high-quality, low roughness film, as shown Figure 6b. The same research group reported In_2_O_3_ field-effect transistor preparation through inkjet printing and two ways of UV curing: UV-vis, UV-laser (Figure 6c). The achieved electron mobility was 8 and 12 cm^2^ V^−1^ s^−1^, respectively [86]. Recently, transition metal dichalcogenides (TMDs), such as MoS, MoSe_2_, and Bi_2_Te_3_, have attracted attention due to their atomic thickness, high surface-to-volume ratio, and tunable bandgap. However, it is challenging to obtain monolayer TMDs films by a widely used method such as exfoliation, thinning, and liquid intercalation [87].

## 3. Printing Technologies

Standard printing technologies can be categorized into two types: non-contact or contact printing. A non-contact printing method uses an ink solution dispensed through nozzles, and printed patterns are defined by moving the stage or nozzle. On the other hand, contact printing technology requires a mask or patterned roll, which makes physical contact with a substrate for printing.

### 3.1. Non-Contact Printing Technologies

#### 3.1.1. Inkjet Printing

**Figure 7:**
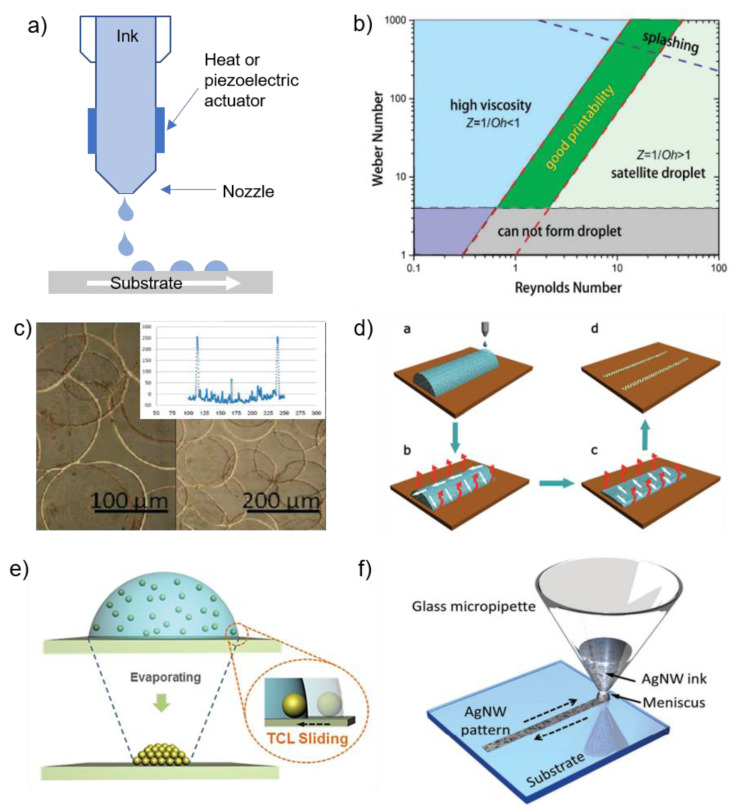
(**a**) Schematic of the drop-on-demand (DOD) inkjet printing; (**b**) the influence of ink properties regarding Weber and Reynolds numbers. Reproduced with permission from reference [6], Copyright 2017, RSC; (**c**) height profiles of printed ring patterns. Reproduced with permission from reference [93], Copyright 2009, CAS; (**d**) schematic representation of inkjet printing of AgNP patterns induced by the coffee ring effect. Reproduced with permission from reference [92], Copyright 2013, John Wiley and Sons; (**e**) schematic of the sliding three-phase contact line (TCL) reversing coffee-ring effect on a low-adhesive substrate. Reproduced with permission from reference [94], Copyright 2014, John Wiley and Sons; (**f**) schematic of the direct-printing process. Reproduced with permission from reference [95], Copyright 2018, John Wiley and Sons.

Inkjet printing is an attractive method to fabricate an extensive range of electronics, including wearable devices, TFTs, solar cells, and RFID (Radio-frequency identification). Nanomaterials, in the form of colloidal or chemical dispersion, are deposited through a micro-sized nozzle. Inkjet printing is versatile and available for industrial-level production, involves few numbers of processes, and can control amounts of deposited materials [37,74,88]. Moreover, the contamination of the substrate can be minimized in that it does not need masks and contact with nozzle [4]. There are two approaches in inkjet printing: continuous inkjet (CIJ) printing, drop-on-demand (DOD) inkjet printing. In CIJ printing, the ink stream is forced to ejected through a small nozzle under pressure [89]. CIJ printing is used for coding and marking due to a larger drop diameter. Since CIJ printing is a wasteful process of ink, the unused ink is recycled. This recycling cause contamination of ink, thus it is suitable for material science. Thus, we will focus on the DOD inkjet printing in materials science. In DOD printing, the ink drop ejects only when the controller asks for it. A thermal or piezoelectric actuator is used to eject ink drops from the nozzle head (Figure 7a).

To form constant patterns uniformly during the printing process without aggregation or clogging, the ink solution used for the fabrication of functional material should have properties that are similar to those of standard inkjet printer inks [90]. The general properties include particle size, viscosity, surface tension, and density. The behavior of the ink can be represented with a few numbers of characteristic dimensionless numbers. They are the Reynolds number (Re), Weber number (We) and Ohnesorge number (Oh) which are defined as:

(1) $$Re=\frac{v\rho a}{\eta }$$

(2) $$We=\frac{v^{2}\rho a}{\gamma }$$

(3) $$Oh=\frac{\sqrt{We}}{Re}=\frac{\eta }{\sqrt{\gamma \rho a}}$$

where ρ,η, and γ are the density, viscosity, and surface tension of the ink, respectively, v is the velocity, and a is the characteristic length, typically the diameter of the print head nozzle.

The reciprocal of the Ohnesorge number (Z) is important to measure the printability of inkjet printing. In Figure 7b, two parallel broken lines stand for the 1 < Z < 10 regions where stable inkjet printing is obtained without satellite droplets. At a small Z value, viscous dissipation prevents the drop-ejection. On the other hand, a high value of Z leads to a great number of satellite drops. Thus, modulating ink composition is important to achieve desired viscosity and surface tension, which leads to stable inkjet droplets [40,90]. The coffee ring effect originates from capillary flow induced by the concentration gradient across the drop [91,92]. When one droplet has a three-phase contact line (TCL), the continuous evaporation of the solvent from the edge causes capillary flow. The capillary flow carries the solute from the center of the drop to the edge, which forms the ring-like shape. This phenomenon is often undesirable because it makes it difficult to obtain a uniform pattern and has a strong negative effect on the morphology of the patterns and electrical performance of the PE. There are two ways to deal with this undesirable phenomenon; minimizing the coffee-ring effect or maximize it. Layani et al. exploited the coffee-ring effect to make a transparent conductive film (TCF) (Figure 7c). 

The inkjet droplets make a 2D array of interconnected AgNP rings. The coffee ring effect makes the AgNP droplets to form self-assembled rings. The width and height of the ring arrays were less than 10 μm and 300 nm, respectively. Transmittance and sheet resistance were 95% and 4 Ω sq^−1^, which is better than the conventional TCF made of ITO. The same group reported the ring array of CNT by inkjet printing. After post-treatment with hot nitric acid, transmittance and sheet resistance were 81% and 156 Ω sq^−1^ each [96]. In another example, the conductive grid pattern of AgNPs was realized by the coffee-ring effect (Figure 7d). Inkjet-printed AgNPs on hydrophilic glass substrates [92]. The low contact angle makes the evaporation rate of solution at the edge of the droplet much higher than the center, leading to solvent loss at the edge. The capillary fluids flow of the solvent transports suspended AgNPs from the center to periphery to replenish the liquid at the edge. The contact line of inkjet-printed droplets forms two parallel lines, which were interconnected in the form of a grid. These gird patterns have line resistivity in the range of 2.61 × 10^−3^ to 5.76 × 10^−4^ Ω cm and a transparency of 92% when the printed patterns are thermally sintered at 160–200 °C for 2 h. Contrary to those approaches, Kuang et al. used a hydrophobic substrate, where the droplet receded inward to the center of the droplet by the sliding TCL and finally forms dome shape with high height-to-diameter ratio (Figure 7e) [97].

Inkjet printing of NWs is challenging due to nozzle clogging of NWs that are generally tens of micrometer length. The general rule of thumb of printability is a/50 (a is the size of the printing nozzle), which is much larger than the NW length. However, reducing the length of NWs usually leads to compromise flexibility, transmittance, and electrical performance. There have been many approaches to improve this trade-off. The first attempt was to increase the viscosity of the ink by adding AgNWs to the AgNO_3_ ink [98]. To improve printability and reduce the sintering temperature, the solvent exchange can be used [99]. First, conductive materials were dispersed in IPA, and then IPA was replaced with DI water. Direct printing can solve the nozzle clogging issue (Figure 7f) [95]. Unlike conventional inkjet printing, AgNW ink is loaded into a micropipette by capillary force. Then, the micropipette contacts the substrate surface, moving along a predefined trajectory back-and-forth to drag the meniscus around. Nozzle clogging can be alleviated by using micropipettes with bigger openings. AgNWs, with length up to 40 μm, are successfully printed on various types of substrates.

Inkjet printing of carbon nanomaterials is also challenging. First, graphene dispersion solvents, such as dimethylformamide and N-methylpyrrolidone, have low viscosity (<2 cP), which is not enough to assure good printability. Second, the graphene concentration in these solvents is usually too low to obtain functional films, so multiple times of print pass is needed. Third, graphene is easily aggregated in inks or during the drying process. Fourth, graphene with minimal defects for high conductivity usually requires post-annealing processes, which might damage other components in the device [5,73]. McManus et al. used pyrene sulfonic acid derivative as steric and electrostatic stabilizers, Triton X-100 to decrease surface tension, propylene glycol to increase viscosity. They successfully disrupted the coffee-ring effect, satellite drops, and nozzle clogging. Moreover, this water-based ink is biocompatible, and this preparation method can dissolve a variety of solutes such as WS_2_, MoS_2_, and BN [100]. In another example, graphene is electrochemically exfoliated in DI water. Additionally, high conductivity was achieved (≈3.91 × 10^4^ Sm^−1^), without an annealing process [101]. Graphene/AgNPs composite ink was successfully inkjet-printed on textiles. The patterns have a stable resistivity variation against bending and folding [102]. Inkjet printing has the versatility to print various materials. Several 2D nanomaterials have been used, including MXene, which is a highly conductive, mechanically robust, and controllable transition metal carbide [103,104]. Interestingly, the unique material composition determines the bandgap state of Mxene, which means that MXene can be a conductor or semiconductor [105]. Some of the recent papers demonstrate the use cases of inkjet-printed MXene as a micro-supercapacitor [106], humidity sensor [107], or photonic sensor [108]. Transition-metal dichalcogenides (TMDs), such as MoS [109], and Bi_2_Te_3_ [110], have also been used for inkjet printing. These nanosheet-layered materials are usually liquid-exfoliated to produce printable inks [109]. PANI and PEDOT:PSS are very attractive conductors, since they can be printed not only solely, but also with other nanomaterials [111]. 

Some inkjet printing techniques make it possible to fabricate various 3D structures. An example uses a patented technology, NanoParticle Jetting, which can use metal and ceramic materials for printing [112]. This system utilizes thousands of printing nozzles to fabricate 3D structured patterns. The nozzles travel over the target building area to deposit inks. The build ink typically uses metal or ceramic nanoparticles, and the created pattern has about 10 μm in thickness and 1200 DPI (20 μm) resolution. Another example explores the Nano-Dimension DragonFly system, which shows its potential to replace the conventional circuit manufacturing [113]. This system has two printing heads for printing metal ink and dielectric ink simultaneously. This printing technique makes it possible to build layer-by-layer structures, including interconnectors, through-holes, and complex patterns.

#### 3.1.2. Electrohydrodynamic (EHD) Printing

**Figure 8:**
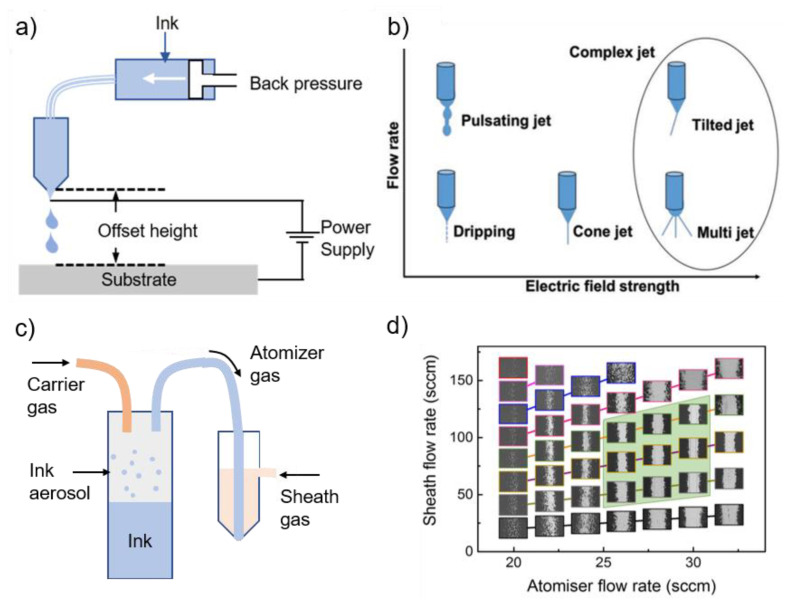
(**a**) Schematic of the electrohydrodynamic printing. (**b**) Diagram depicting different jetting modes as flow rate and electric field are changed. Reproduced with permission from reference [4], Copyright 2019, John Wiley and Sons. (**c**) Schematic of the aerosol printing. (**d**) The influence of two gas flows online morphology. Lines printed at the same focus ratio (FR = sheath flow rate/atomizer flowrate) are grouped. A clear operability window (highlighted green) can be seen for atomizer flowrates between 26 and 30 sccm, with focal ratios between 2 and 4. Reprint is in accordance with the Creative Commons Attribution 3.0 International License [132].

The EHD printing method refers to a technique that uses electric fields, rather than thermal or acoustic actuation, to create fluid flow. Ink is ejected by generating a high electric field between the nozzle and the substrate [11,114,115]. EHD printing produces much smaller droplets than conventional inkjet methods [116]. Park et al. achieve a minimum diameter of 240 ± 50 nm with a nozzle with a diameter of 300 nm [114]. EHD printer is comprised of a pressure regulator, ink chamber, nozzle tip, substrate, translational stage (Figure 8a) [4]. The printing conditions are primarily controlled by backpressure, offset height, and applied voltage. The type of applied voltage defines the mode of ink ejection from the nozzle. DC voltage results in an intact jet, whereas AC voltage at different frequencies and functions define the DOD mode of the system [11]. The applied voltage between the nozzle and the substrate decides the strength of the electric field. The relationship between the flow rate and the electric field strength is shown in Figure 8b. When the flow rate and electric field are well optimized, the stream with the “cone-jet” mode is ejected. However, “complex jet” mode comes along with an excessive increase in the electric field strength [117,118]. EHD printing of conductive features in a continuous cone-jet mode is challenging, as the charged nozzle and the grounded substrate can be electrically connected through the conductive ink. This leads to discharge and termination of the printing process [119]. To enhance the resolution and performance, a variety of nozzle designs are reported: multi-nozzle [120], multi-hole nozzle [121], co-axial nozzle [122,123], and tip-assisted nozzle [124]. Recently, tip-assisted EHD printing can reduce the starting and working voltage, which improves the stability and uniformity of the jet.

EHD printing of metal nanomaterials shows higher resolution than other printing methods. The grid width of less than 10 μm was achieved with AgNPs ink for TCE. The sheet resistance of 4.87 Ω sq^−1^ and the transmittance of 81.75% was realized after annealing at 200 °C under near-infrared light [125]. Cui et al. reported EHD-printing AgNWs on various types of substrates such as PDMS, PET, glass, letter paper, nanofiber paper. The printed patterns have high conductivity as high as ~5.6 × 10^6^ Sm^−1^ [28]. Moreover, the patterns were printed on gloves, showing stable performance under bending, stretching, and twisting. Printed line width is determined by the parameters, including ink viscosity, AgNW concentration, standoff, printing speed, voltage, nozzle size, and pressure. To improve electrical conductivity, metal nanowires have been widely used. AgNWs/PEDOT:PSS nanostructured thin films were printed by using an EHD printer [125].

In addition, the EHD printing method offers a promising strategy for fabricating hierarchical fibrous 3D patterns. He et al. reported solution-based EHD 3D printing techniques with CNT. Poly (ε-caprolactone) and polyethylene oxide were chosen to build the microscale scaffold structure. The printed fiber was about 10 μm in diameter and showed biocompatibility with a cell culture experiment [115]. In another example, low AgNP viscosity ink enabled self-sintering, one-step EHD printing process. The ink solvent evaporated immediately before contacting the substrate as the charged AgNPs formed aggregated AgNP junction and 3D structure. Submicron-scale of 3D structures were obtained with an aspect ratio of 35 [126].

#### 3.1.3. Aerosol Printing

In aerosol printing (Figure 8c), the functional ink is aerosolized and entrained in a carrier gas (atomizer) such as N_2_, He [4,126,127,128,129,130]. There are two options to generate aerosols via the pneumatic and ultrasonic atomizers. The aerosol stream is directed to a print head, where it is aerodynamically focused by a coaxial sheath gas flow [131]. The jet in aerosol jet printing is comprised of many droplets with a diameter of 2–5 μm. The ratio of the sheath gas flow rate to the carrier gas flow rate is the key parameter in aerosol printing, the focusing ratio, which defined as:

(4) $$focusing\;ratio=\frac{sheath\;gas\;flow\;rate}{carrier\;gas\;flow\;rate}$$

By increasing FR, the deposition quality and the more distinct printed line can be obtained. However, a further increase in FR can degrade the printed pattern (Figure 8d) [127,128,132]. In addition to FR, the influence of substrate temperature on the line morphology was studied for three different substrates: silicon, glass, and polyimide. The results indicated that the differences between the morphology of the line are not distinct at low temperatures. However, as the substrate temperature is increased, the differences become apparent [132].

Due to the high distance between the nozzle head and the substrate, aerosol printing is compatible with non-planar substrates [127,130,132,133]. AgNW conductive traces are deposited on a 3D-printed rough, non-uniform structure with aerosol-assisted atmospheric pressure plasma-based printing [134]. This technique can also be applied to other substrates, such as cellulose paper and flexible cellulose acetate. Ag interconnects were printed on the outer sidewall of a hollow plastic pillar with 22 μm in width [129]. Moreover, aerosol printing can also build a 3D structure; AgNPs, dispersed in EG and DI water, were successfully printed by the aerosol printing method to build high intricate microscale 3D networks, such as micro scaffolds/micro lattices [135]. 

By adding carbon-based materials, such as graphene or CNT, the conductivity of the printed patterns can be improved. Carbon materials fill the gaps and defects, such as granular boundaries between metal nanoparticles [136]. Zhao et al. formulated CNT/AgNP composite inks for the aerosol printing system. With a few additions of CNT (0.15% wt), the conductivity of the printed line was 38% lower than pure silver lines. In another example, the addition of carbon nanomaterials improved flexibility and mechanical robustness. Jabari et al. reported a graphene/AgNP ink for the aerosol printing system, where the printed patterned were subjected to bending over a tube with a 5.5 mm radius of curvature and up to 1000 bending cycles [136]. After the bending cycles, the resistance of the printed patterns remained relatively constant.

### 3.2. Contact Printing Technologies

#### 3.2.1. Screen Printing

**Figure 9:**
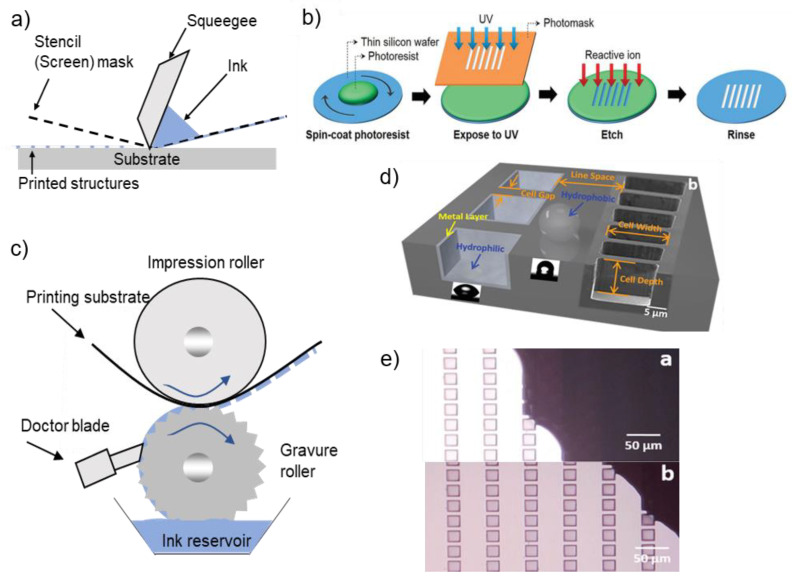
(**a**) Schematic of the screen printing. (**b**) Schematic of a typical microfabrication process for creating thin silicon stencils. Reprinted with permission from reference [70], Copyright 2015, John Wiley and Sons; (**c**) Schematic of the gravure printing. (**d**) Illustration of the wettability contrast between the hydrophobic surface on the land and hydrophilic surface in the cell. (**e**) Time-sequence optical images showing the ink loading process by guiding a drop of an 80-wt% glycerol–20 wt% water solution over the pattern toward the upper right corner. Reprinted with permission from reference [147], Copyright 2015, John Wiley and Sons.

The screen-printing method is one of the most mature and versatile methods with fast processing speed, high efficiency, and low cost. A typical screen printer has a simple setup, including a screen, squeegee, press bed, and substrate (Figure 9a). The ink poured on the squeegee is pressed through the screen, resulting in its transfer through the stencil openings to the substrate under the screen. Flat screens can be replaced with rotary screens to improve printing speed and efficiency [11]. Solution viscosity, printing speed, angle, and morphology of the squeegee, the gap between screen and substrate, mesh size, and materials can affect the print uniformity and resolution [4,137,138,139,140]. The disadvantage of the screen printing method is in relatively low resolution, which is in the range of 50–100 µm commonly [11,141]. To improve the resolution, Hyun et al. used a silicon stencil (Figure 9b). The fine stencil pattern was developed through photolithography and reactive ion etching. The opening of the stencil was as small as 5 µm, resulting in the printed line resolution below 40 µm [70].

Metal flakes or particles are not cooperative to screen printing with the linewidth below 70 µm, which results in high shadow loss [142]. Li et al. optimized AgNW loading (2 wt%) to deposit the printed pattern on the PDMS substrate. With 73% of transparency, they maintained flexibility and stretchability with good conductivity (~1.74 × 10^5^ S/m). Tam et al. reported a Cu paste for screen-printing; they dispersed CuNP in EC and terpineol to make a high concentration (1M) of Cu paste, with good control of the size distribution of nanoparticles (12 to 99 nm) [143]. The electrical resistivity of patterns after a low temperature (120 °C) sintering treatment was approximately 5.8 × 10^−5^ Ω cm. Recently, screen-printable Cu metallic oxide decomposition (MOD) ink was reported [144]. They printed Cu traces with Cu ink comprising a MOD compound, conductive filler, and binder, followed by thermal and IPL (intense pulse light) sintering. The conductive traces showed oxygen stability, mechanical robustness, good electrical performance, and direct solderability on flexible PET substrates. Additionally, SWCNT TFT on a Si wafer was fabricated with a screen-printed Cu source and a drain electrode, which performed 12−15 cm^2^ V^−1^ s^−1^ and current on/off ratio of ∼10^5^. 

To enhance the resolution and reliability of the screen-printed NPs, the adhesion mechanism between AgNPs and the substrate were investigated [145]. The interfacial fracture energy of screen-printed AgNP films on the silicon substrate was quantified to understand the adhesion mechanism. The interfacial fracture energy is significantly influenced by the surface morphology and the number of organic residues at the interface. The optimum sintering temperature was 250 °C, where the interfacial fracture energy is at its zenith, due to the increase of surface roughness and the presence of a sufficient amount of organic residues at the interface. To screen-print carbon nanomaterials with high conductivity, it is important to remove the binder or surfactant component of the ink from the printed patterns. Wu et al. fabricated a screen-printed temperature sensor with flake graphite (FG) /CNT/PDMS composite. By optimizing the mass ratio of FG to CNT (4:1), the temperature coefficient of resistance value was maintained at the level of 0.028 K^−1^ with good sensibility and linearity [146].

#### 3.2.2. Gravure Printing

Gravure printing is a widely used printing technique that can offer high-throughput R2R patterning of materials at high speed [11,148]. Gravure printing relies on surface tension transfer of ink from small engraved cells on the gravure roller to the substrate [149]. Proper contact between the roller and the substrate is important to print the patterns. The cells on the roller are continuously refilled, bypassing the ink reservoir, as the doctor blade (Figure 9c) removes excess ink. Solution properties and cell width/depth ratio is a key parameter which determines the printing quality. The low viscosity ink can enhance the printing speed [150]. For efficient transfer of the ink, the proper ratio of the width of the cell to depth is usually 7–8, which facilitates uniformity of the patterns and prevents the ink from invading adjacent patterns. In addition, cell spacing is also an important factor, which is usually 1.06–1.4 [151]. Zhang et al. improved the printing resolution with the use of a wettability contrast [147]. To facilitate the wettability contrast, the gravure pattern consists of hydrophilic cells and hydrophobic lands, where the cell was deposited by a nickel layer, and the Si lands were treated by trichloro (1H,1H,2H,2H-perfluorooctyl) silane (Figure 9d). The water contact angles were 30° (Nickel) and 110° (trichlorosilane), respectively. This contrast successfully removed excessive ink without using a doctor blade (Figure 9e). Continuous lines with 1.2 µm in width and 1.5 µm in spacing were achieved.

AgNP is often used for gravure printing. As an alternative to the conventional thermal sintering, the laser sintering treatments after printing attracted interests [152,153,154]. Lee et al. reported AgNP film on a flexible PET substrate with the R2R gravure printing process, followed by the laser sintering process that can replace the conventional thermal annealing process [155]. With this advanced post-treatment process, the resultant AgNP film restores bulk state electrical conductivity very swiftly, without any thermal damage to the flexible substrate beneath the film. Park et al. investigated the gravure printing of AgNW using intaglio trench patterns on a PET film substrate [155]. By optimizing the printing speed and pressure, the printed line resistance for a 450 μm of line width dried at 90 °C was 32 Ω mm^−1^ with a 95% of transmittance and a 100 μm spacing between the printed lines. Huang et al. tailored AgNW ink properties to improve the performance of gravure-printed devices [156]. The AgNW ink, which contains a low solid content (5.0 wt%), had a high viscosity (20 Pa·s) as well as good rheological behavior suitable for gravure printing. The resultant printed pattern showed 5.34 × 10^4^ S cm^−1^ after a low thermal annealing temperature of 150 °C. R2R gravure printing for AgNW is also investigated. AgNWs network was R2R printed on the PET web, where the printing parameters (PET web tension, roll speed, and line speed) were optimized for better printing performance [157]. After the printing process, the coated film was moved to the internal heater for the drying process. During the laser-induced plasmonic welding process, the laser was scanning to make the local fusion of AgNW and improve Ag network contacts. As a result, thermally activated isolated Ag atoms flow over the nano junctions and recrystallize to solder point. Sheet resistance ~5 Ω/sq at high transparency (91% @λ = 550 nm) was achieved for the applications in optoelectronic devices.

SWCNT-TFT based active matrices (AM) with a 9.3 ppi (points per inch) resolution were investigated with a fully R2R gravure printing technique on the PET web [158]. After laminating a pressure-sensitive rubber sheet on the printed AM, the printed AM can be used as a multi-touch sensing sheet. Gravure printing of hybrid MoS_2_ nanoflowers@sulfonated reduced graphene oxide (S-rGO) interdigitated electrode showed a highly porous pattern, which leads to a micro-supercapacitor on a PI substrate [159]. This optimized structural design, which is in larger active surface area, has better accessibility of electrolyte ions compared to conventional vertical sandwich structures.

#### 3.2.3. Flexographic Printing

The flexographic printing method was developed for thin, uniform layers, providing better integrity and narrower pattern edges than gravure painting [141,160]. Flexographic printing is a printing process that employs a rubber or polymer plate [11,161,162]. The inked areas of the anilox cylinder contact with the engraved patterns on the plate cylinder. The quantity of ink to be translated to the substrate is primarily controlled by anilox roll. The doctor blade removes the excessive ink. Flexographic printing can print a variety of ink types (solvent-based, water-based, electron-beam curing ink, UV curing ink), as well as a variety of substrates type (porous, non-porous, flexible, rigid) [10,11]. Kim et al. investigated the nanoporous stamp, which was comprised of polymer-coated aligned CNTs [10]. The ink, well-matched with the pattern of the structures on the stamp with high fidelity, was transferred onto the substrate. A variety of nanoparticle inks (Ag, ZnO, WO_3_, and CdSe/ZnS) was printed in diverse patterns on both rigid and flexible substrates. The resolution patterns with a width of 20 μm were achieved with 0.2 m/s printing speed.

### 3.3. Strategies for High-Throughput Printing Devices (PEs)

When printed wearable electronics are manufactured, parameter optimization is crucial for high-throughput fabrication as well as the development of printing technologies. To make high-throughput PEs, there are multiple parameters to be considered, including resolution, uniformity, flexibility, stretchability, and durability. High-resolution and uniform patterns can reduce the size of the device while maintaining performance. The flexible and stretchable patterns can give users comfortable wear on the skin as well as conformal contact for the high-fidelity recording of physiological signals. Lastly, good durability ensures long-lasting, reliable performance, and robustness of PEs against delamination, bending, and stretching. Table 2 summarizes the key parameters to consider in the printing of wearable electronics.

#### 3.3.1. High-Resolution Patterning

High-resolution patterning allows for the design of densely packed and sophisticated electronics. Moreover, these patterns can enhance device performance via larger bandwidth and higher on/off speed for transistors [40,141].

For non-contact printing technologies, coating substrates to control the morphology of the substrates was reported. Lessing et al. modified the surface free energy of papers by coating them with fluoroalkyls, which render substrates omniphobic. The coated paper enlarges the contact angle of the ink drops, minimizing the spreading degree and width of printed lines [163]. In another example, a fluorine-treated polyimide film was fabricated. It has a porous structure, which successfully inhibits the spreading of AgNP ink [164]. Wu et al. made a pre-patterned hydrophilic 3D microstructure on substrates. The asymmetric dewetting of the droplet’s TCL resulted in diverse, controllable 3D morphologies of ink droplets [165]. Recently, Liu et al. fabricated bio-inspired 3D printed micro-arrays, which show no residue adhesion, tunable, and absolute adhesion of micro liquid droplets [166]. The coffee ring effect is a crucial parameter to consider fabricating high resolution and uniform patterns. By manipulating the fraction of octane in the ink, the capillary flow and the inward Marangoni flow are balanced, which results in constant thickness across droplets [167]. In EHD printing, reducing nozzle size or novel designs of tips were reported to enhance printing quality [114,124]. In aerosol printing, the optimization of the focal ratio and line geometry is important to make distinct, narrow lines [131].

For contact printing technologies, many endeavors are devoted to making more sophisticated stencils or engraved cells [11,141]. Hyun et al. introduced a thin silicon stencil made from conventional lithography techniques [70]. In addition, a method that makes a wettability contrast between the surface of the cell and land was reported (Figure 9d,e) [147]. This contrast makes the ink stay not on the land but in the engraved cells.

#### 3.3.2. Uniformity

The uniformity of printed lines is essential in inkjet printing, since inkjet printing does not require stencils or masks guiding the ink spreading. The coalescence of consecutive droplets without defects ensures the uniformity of printed patterns. The process of droplet coalescence can be described with a few stages [40,169]: (1) rapid merging of the separated drops, (2) slower rearrangement of the liquids, and (3) mixing of the fluids. By adjusting the spacing of the consecutive droplets and optimizing the delay time of ink ejection, the deposition of the morphology of printed patterns can be controlled [170,171]. By manipulating surface tension of the substrates, different dynamic wettability of ink droplets to substrates was realized [9,171]. Soltman et al. reported solvent evaporation, which was induced by the temperature of substrates. A cooled substrate suppresses edge evaporation and eliminates the coffee ring at the droplet’s edge [170].

#### 3.3.3. Flexibility/Stretchability

Unlike the conventional rigid substrates, flexible substrates, such as plastics, papers, and elastomers, are widely used to develop wearable electronics. Plastics can be used in long-time applications, whereas paper can be used in one-time and disposable applications. Plastics have hydrophobic surface and high mechanical strength, which ensure good printability, but they often become gossamer during the high-temperature sintering process, because of their intrinsic low glass-transition temperature. To overcome this disadvantage, room temperature sintering methods have studied, some of which are listed on 2.4 Post-printing treatment. Owing to the physical-chemical properties of a cellulosic paper, papers are disposable, inexpensive, biodegradable, and can be rolled or folded into diverse configurations [176]. To prevent overspreading of ink into paper substrates, various paper textures and coatings are reported: employing photo paper [177,178], modifying paper to hydrophobic [179,180,181], reducing the surface roughness [182,183], and coating paper [163]. 

Elastomers are the best choice for the substrate, considering both flexibility, and stretchability. Silicon elastomers, such as Ecoflex, Sylgard, Dragon Skin, and Silbione, are biocompatible in general, and they are very compliant with maximum elongation up to 900% [18]. Conformal contact of such elastomers onto target areas can be achieved in the engineering of thin-film formation. Elastomers can be embedded with sensing nanomaterials to improve electrical performance while maintaining favorable mechanical properties. Chung et al. reported an embedded stretchable silver electrode, which was printed on PDMS substrates [184]. The same group developed a highly stretchable strain sensor which utilizing the negative strain-dependency in the electrical resistance of the magnetically patterned and arranged nickel composite. The exposed silver-covered electrode on the PDMS substrate shows stable conductivity up to 100% tensile strain [26]. Open-mesh structures or serpentine structures show conformal contact for gathering human biopotentials in the field of FHE. With the use of open-mesh structure, conformal skin contact was achieved to measure high-quality electroencephalograms (EEG) [29]. Serpentine structure of AgNP-printed sensor, encapsulated by Si elastomer, accommodates a high stretchability of up to 250% of radial stretching and a cyclic bending [23].

#### 3.3.4. Durability

The mechanical durability is another important aspect of the fabrication of FHE, which is often under the condition of persistent, complex strains. Dynamic durability represents the perseverance of stable electrical performance and mechanical integrity under long-term and consistent bending or stretching movements, such as when contacted with the human skin [40]. Strategies to prevent degradation of the performance of FHE will be discussed.

Degradation often emerges during harsh conditions such as high temperature or high pressure. During the thermal sintering process, plastic and paper substrates can easily be damaged. By optimizing the sintering temperature with the adhesion mechanism of films, mechanically robust electronics application can be achieved [145]. In other approaches, sintering treatments without thermal processes, such as UV curing, plasma sintering, phonic sintering, and microwave sintering. Adding binders such as ethylcellulose to the ink is also a good option to prevent cracks [16]. However, binders can increase the viscosity of the ink, especially CNT/graphene. Moreover, adding non-conductive binders might require extra sintering processes. Reducing the tensile strength provides another option. Guo et al. reported flexible, foldable, and stretchable conductors. By laminating another layer of PET on top of the Cu layer, PET/Cu/PET sandwich structure can effectively shift the stress-neutralization plane into the Cu layer. With this structure, the film stress in the Cu layer is shifted to shear stress, reducing the cracks [185]. The remarkable self-healing of the biological system can be applied to developing new repair strategies [186]. Capsule-based self-healing materials released the encapsulated healing agent when the printed device was mechanically damaged. The healing agent locally dissolves the binder and makes the rearrangement of nanomaterials such as AgNPs, which leads to restoring the electrical conductivity [174]. In another example, Kee et al. reported a self-healing, organic thermoelectric film [175]. The surfactant matrix entangles with PEDOT:PSS chain can flow from both sides to the void area and finally form physical contact with each other. The hydrophilic functional group of Triton X-100 is effective to form entangled formation with hydrophilic PEDOT:PSS, where liquid and viscous Triton X-100 can act as the soft viscoelastic matrix. Consequently, the polyethylene-oxide-based hydrogen-bonding site of Triton X-100 can restore the cut parts at the interface. As a result, the thermoelectric properties of the composite films are recovered. The addition of conductive textiles provides electrical stability and durability of patterns. Tadesse et al. reported conductive textiles that maintain conductivity during repeated laundering [187]. In this work, a polyamide PA/lycra fabric is immersed in PEDOT:PSS with PU dispersion. This elastic conductive fabric shows good conductivity until stretched 650%. The added PU offers maintained conductivity and durability against repeated laundering.

## 4. Applications

### 4.1. Nanomaterial-Enabled Sensors

In this section, we summarize various types of wearable sensors that are fabricated by printing technologies. Table 3 shows the summary of wearable sensors, used materials, printing methods, and performance.

#### 4.1.1. Temperature Sensors

**Figure 10:**
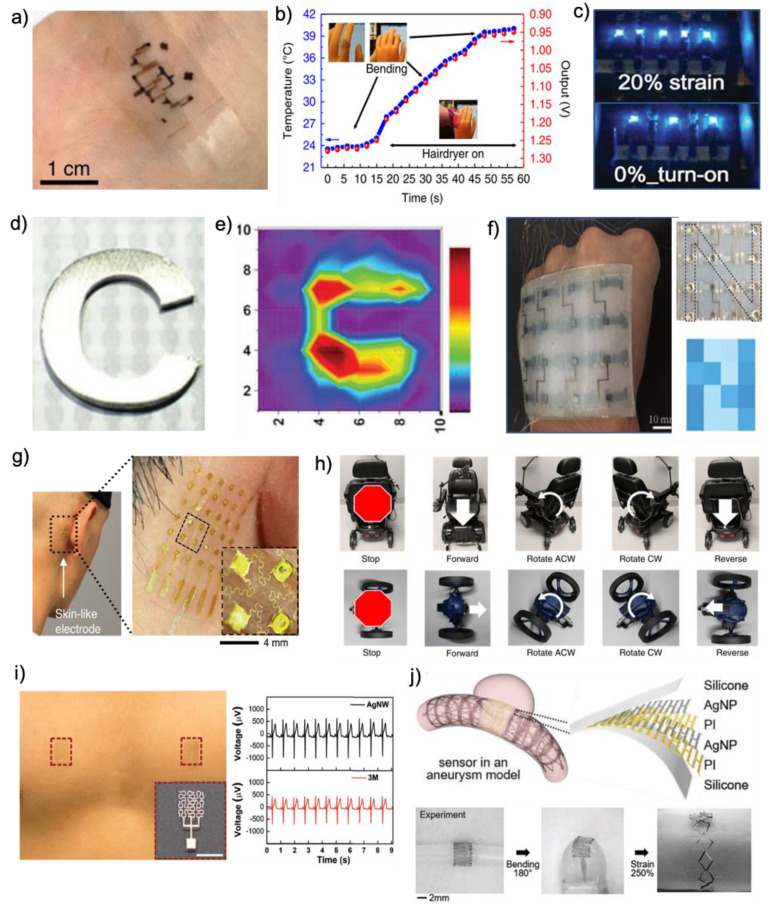
(**a**) Conformal wearable temperate sensor; (**b**) stable performance of the temperature sensor during bending. Reprinted with permission from reference [25], Copyright 2018, Springer Nature; (**c**) luminance change of strain sensor with tensile strain. Reproduced with permission from reference [26], Copyright 2014, John Wiley and Sons; (**d**) top view of the “C” letter positioned over the pressure sensor array and the corresponding pressure signal distribution. (**e**) photograph of the strain sensor array is attached to the back of the hand. Reprinted with permission from reference [57], Copyright 2018, John Wiley and Sons; (**f**) an image of the sensor array pressed by 10 cylinders with N-shaped and the corresponding capacitance signals of the 16 pixels. Reprinted with permission from reference [24], Copyright 2019, John Wiley and Sons; (**g**) a skin-like electrode with an open-mesh structure; (**h**) control of machines via classified EEG signals, including a wireless electric wheelchair with five classes (no action, forward, rotate anticlockwise (ACW), rotate clockwise (CW) and reverse), a wireless vehicle with the same commands as the wheelchair. Reproduced with permission from reference [29], Copyright 2019, Springer Nature; (**i**) an image of printed AgNW dry electrocardiogram (ECG) electrodes and ECG signals collected from the electrode. Reproduced with permission from reference [28], Copyright 2018, RSC; (**j**) illustration of the fabrication of an implantable flow sensor in an aneurysm model and its explosive view. The below image is an experiment of the stretchability of the sensor. Reprint is in accordance with the Creative Commons Attribution 4.0 International License [23].

Body temperature is one of the crucial indicators in human health monitoring, and can be used as a harbinger of an abnormal condition of the body. Conventional thermometers with periodic measurement can easily measure it, but real-time monitoring of the body temperature is required to prevent acute diseases, such as heat stroke or congestive heart failure. In this application, conformal contact on human skin, high precision, fast response, good repeatability, and long-term stability are required. The wearable temperature sensors should function in the temperature range from 25 to 40 °C, in which all values of human temperature conditions are covered [103]. In addition, high accuracy with a clinically desired resolution of 0.01 °C is also necessary. Most wearable temperature sensors detect the change of electrical characteristics of sensor components. Harada et al. reported a fully printed flexible temperature sensor with CNT ink and PEDOT:PSS. This sensor can detect temperature between 21 and 80 °C, with a sensitivity of ~0.25%/°C (ΔR(=R-R0)/R0 (%)) [190]. In another example, Zhu et al. reported a temperature sensor which is high conformal on human skin even on joint areas, which is shown in Figure 10a [25]. Multiple TFTs based on SWCNT with dynamic differential circuit design demonstrated −24.2 mV °C^−1^ in the temperature range of 12–55 °C (Figure 10b), and minimize strain-induced error, measuring the temperature within ±1 °C, uniaxial strain range of 0–60%. 

#### 4.1.2. Strain/Pressure Sensors

Strain sensors convert external mechanical deformation into an electrical signal of sensing materials [191]. There are many types of sensing principle: piezoresistivity, piezoelectricity, capacitance, percolation network, crack propagation, resonant frequency shift, and triboelectricity [103]. A strain sensor with a higher gauge factor has better sensibility, with which the subtle motions of the subject can be detected. Pressure sensors can use capacitive or resistive-based measurements, depending on what the sensing material will be used [18]. High-pressure sensitivity with a low detection limit is desirable for the pressure sensors. Both of the sensors should have a low-temperature coefficient of resistance, which is a value that denotes how the sensitivity of sensors is responsive to temperature change. A sensor that has negatively strain-dependent electrical resistance change was reported [26]. This property originates from the magnetically patterned and arranged nickel composite. Inkjet-printed Ag film provides high stretchability to an electrode, which can be easily elongated with very low initial resistance (20 Ω) up to 100% tensile strain. As shown in Figure 10c, three pixels are turned on constantly; two hidden pixels are turned on additionally when the electrode is stretched. Yang et al. reported a permeable pressure sensor based on a layer-by-layer structure of porous nanofiber membranes and AgNW (Figure 10d,e) [57]. The pressure sensor has a sensitivity of 4.2 kPa^−1^, the fast response time (<26 ms), and the low detection limit (1.6 Pa). An all-inkjet-printed, bimodal sensor that can measure pressure and strain simultaneously was reported (Figure 10f) [24]. AgNPs suspension was used as a conductive ink. The top electrode is paper, while the bottom electrode is polyethylene naphthalate (PEN). Two electrodes are combined to form a capacitive pressure sensor, while the bottom electrode serves as a strain sensor. The pressure sensor has low-pressure detection of 2 Pa with fast response time (126 ms), while the strain sensor has high GF (4000) and fast response time (154 ms).

#### 4.1.3. Electrochemical Sensors

Biofluids such as sweat, saliva, and tears can provide medical information without collecting a blood sample. Noninvasive wearable electrochemical sensors have been developed to tracking such biomarkers [17,192,193]. Electrochemical sensors should have high sensibility, reliability, fast response time, and precise selectivity with a wide detection range of concentration of biomolecules. Screen-printed electrodes can be easily fabricated in diverse shapes and sizes and applied to various biological target analytes [16]. Furthermore, as discussed in the printing technologies section, screen-printing is reliable, versatile, and fast processing speed at low cost. Considering these advantages, screen-printing has been used to fabricate low-cost, non-invasive, real-time monitoring electrochemical sensors. A temporary tattoo-based electrochemical sensor was reported by Wang’s group [194]. This flexible enzymatic biosensor shows chemical selectivity toward lactate up to 20 mM with linearity and resilience against mechanical deformation from the wearer’s movement. The same group developed a glucose sensor that detects glucose from human perspiration [189]. This sensor showed the quantification of glucose with a broad working range (33 μM–0.9 mM), along with an excellent correlation with a commercial glucose sensor.

#### 4.1.4. Electrophysiological Sensors

Electrophysical sensors can detect the changes in voltage in which biological cells and tissues produce [103,195,196]. Among these biopotentials, electrocardiogram (ECG) provides detailed information on the ventricles and atria for heart activities to diagnose and manage cardiovascular diseases [17,103]. Electromyogram (EMG) measures electric potential generated by the tissues and nerves, which is useful to diagnose neuromuscular disorders such as Parkinson’s disease, Duchenne muscular dystrophy, and spinal muscular atrophy [197]. Electroencephalogram (EEG) monitors the electrical activity of the brain, which is useful to diagnose brain-related diseases such as sleep disorder, epilepsy, amnesia [17]. Electrooculography (EOG) captures biopotentials generated by the corneo-retinal dipole potential changes from eye movements. The biopotentials collected from various types of wearable sensors show a wide range of frequency scale (1 Hz ~ a few kHz) and voltage range (1 μm ~ 1 V) [103]. The detection of such subtle electrical signals from the skin surface requires high electrical conductivity and intimate skin contact of sensors that are directly related to good signal-to-noise ratios and minimized motion artifact. Following this signal collection, a signal-processing model is required to filter out undesirable noise and extract valuable data from the raw data.

### 4.2. Printed Electronics

Nanomaterials enabled the development of soft, conformal, and stretchable sensors for high quality, real-time monitoring of human health. With these sensors, high-performance and miniaturized FHE has been developed for a wide range of healthcare applications. In this section, we will provide an overview of the novel and representative FHE technologies, which resolve the challenges of conventional healthcare devices that are rigid, bulky, and heavy.

#### 4.2.1. Prosthesis

A fully portable and wireless, flexible scalp electronics system, comprised of a set of dry electrodes and a flexible membrane circuit, was developed (Figure 10g,h) [29]. The aerosol-printed skin-like electrode is mounted on mastoid (Figure 10g), and the other three flexible conductive electrodes are attached on the hairy scalp (occipital lobe). This minimized wireless electronic system offers maximum comfort and minimal set-up time, requiring only two channels to capture the state visually evoked potentials (SSVEP). Moreover, the system included a deep-learning algorithm, convolutional neural networks for universal, multi-channel classification of SSVEP. The feasibility of this device was successfully demonstrated via real-time and wireless controls of external machines with SSVEP (Figure 10h). Multiple external devices, including a powered wheelchair, a wireless vehicle, and a PowerPoint presentation, were successfully controlled by gazing LED stimuli in front of the subject’s eyes, about 0.8 m away from their head at eye level.

#### 4.2.2. Healthcare 

An AgNW-based wearable health monitor was reported (Figure 10i). With EHD printing, AgNW-based dry ECG electrodes without the electrolytic gel layer were realized. The fractal pattern of the Greek Cross on the PDMS substrate effectively releases the local strain under mechanical deformation on the skin. This sensor measures high-quality ECG signals that are comparable with the conventional wet electrodes. 

#### 4.2.3. Implantation

Cerebral aneurysms result from weakened sections of blood vessels. The continuous incoming blood flow to the ballooned section of the vessel may cause rupture or even more serious damage [198,199]. To address this issue, a highly stretchable nanomembrane system for wireless monitoring was developed, which can conformally be integrated onto a medical stent and deployed into a blood vessel via the conventional catheter procedure [23]. The aerosol-printed, nanostructured sensor shows an exceptional stretchability of 250% and flexibility of 180° bending (Figure 10j), which captures the device’s ability to be placed around highly contoured and narrow cerebral arteries. Furthermore, the utilization of an inductive coupling method with two external coils enables a batteryless, wireless detection of the printed sensor.

## 5. Conclusions and Outlook

Recent progress in printing technologies and functional nanomaterials has enabled breakthroughs in various types of sensors and electronics, particularly in wearable FHE systems. This review article delivers the summary of nanomaterial properties, synthesis of nanomaterials, printing strategies, and use cases in wearable and implantable applications. Nanomaterials have improved the mechanical properties of wearable electronics, while maintaining favorable electrical performance. Advances in printing technologies and material processing techniques allow for exceptionally small form factors, even with the integration of high-performance multifunctional sensors and electronic components. The nanomaterial-enabled FHE systems have enabled advanced portable health monitoring, real-time disease diagnosis, and long-lasting machine interfaces.

For next-generation FHE, we still need continuous improvements in scalability with the maintained printing quality. For example, a scalable R2R process can offer an industrial level printing while requiring a higher resolution of printed patterns. Another critical factor to consider is the reusability and reliability of FHE. As we have more matured fabrication technologies, a reliable device packaging needs to be further investigated for long-term, continuous, and multiple uses of the FHE.

In addition, the seamless integration of multiple functional materials will offer enhanced FHE performance and mechanical durability. For example, the hybrid integration of electronics with textiles can provide better breathability, wearability, and reliability. Incorporation of FHE with papers can make a low-cost, disposable system for single-use cases. Furthermore, the addition of emerging self-healing materials along with wireless powering units will offer a long-term usable, reliable health monitoring system.

Lastly, the continuous development of thin, high-performance electronic chips is required to maintain the device’s flexibility and stretchability, while offering high-fidelity and wireless monitoring of physiological signals on the skin or internal organs.

In summary, we believe that the consideration of the existing challenges in printed electronics will provide new opportunities to utilize the FHE for more practical healthcare applications in daily life.

## Figures and Tables

**Table 1:** Properties of conductive nanomaterial inks for printed electronics.

Materials	Resistivity/Conductivity	Solvent/ Binder	Particle Size	Sintering Condition	Metal Concentration	References
AgNW	44.0 Ω/sq	water	L = 8.63 to 29.8 m D = 56.6 to 84.2 nm	120 °C, 5min	0.2 wt%	[47]
AgNP	9.18–8.76 × 10^−8^ Ω m	EG ^1^, ethanol, and water	19.1 ± 1.7 to 22 ± 1.8 nm (depending on PVP/AgNO_3_ ratio)	160 °C, 30 min	10–15 wt%	[44]
AuNP	16 μΩ cm	xylene	3 nm	IR-sintered for 10–15 s	15 wt%	[48]
CuNW	19.8 Ω/sq	diethyl ether	L = a few hundred micrometers D = 45 ± 5.7 nm	200 °C, 30 min under N_2_ atmosphere	0.063 M	[49]
CuNP	5.5 μΩ cm	toluene	42.3 to 108.3 nm	350 °C, 4 min	30 wt%	[33]
95% CuNP/5% CuNW	22.77 μΩ cm	10:1 DEG ^2^, PVP	Cu NWs (150 ± 50 nm in diameter, 1−2 μm in length) Cu NPs (20−50 nm in diameter, oxide thickness > 2 nm	Flashlight sintered	53.5 ^3^ wt%	[13]
PdNP	2.6 μΩ cm	toluene	-	200 °C, 4 min	>14 wt%	[37]

^1^ Ethylene glycol, ^2^ Diethylene glycol, ^3^ Values calculated from the reported data.

**Table 2:** Key parameters for the manufacturing of wearable electronics.

Category	Remarks	Strategies	References
Resolution	High resolution is required to enhance the degree of integration and electrical stability.	A low surface energy coating	[163]
		Make pre-patterned structure on substrate (inkjet)	[165,166]
		Reverse coffee-ring effect (inkjet)	[97,167,168]
		Reduce nozzle size (EHD)	[114,124]
		Introduce tip-assisted nozzle (EHD)	[124]
		Manipulate focus ratio(aerosol)	[131]
		Finer screen mask (screen printing)	[70]
		Small engraved cells (gravure)	[11,141]
		Make wettability contrast between cell and land(gravure)	[147]
Uniformity	Uniform ink deposition is required to increase the electrical stability and reliability of the electronics.	Adjust drop spacing	[8,169,170]
		Optimize delay time (inkjet)	[169,170]
		Manipulate the ink droplet’s surface tension	[9]
		Adjust viscosity/surface tension ratio	[171]
		Control evaporation speed of solvent in the ink	[170]
Flexibility	High flexibility is important to decide the application spectrum of devices and allows intimate contact without mechanical failure.	Use paper or plastic substrates	[88,163,172]
		Utilize open-mesh, serpentine structures	[173]
Stretchability	High flexibility is required to conformal contact of the device to human skins and long-time stability of the device.	Introduce elastomer substrates	[23]
		Utilize open-mesh, serpentine structures	[23]
Durability	Mechanical robustness against long-time and continuous bending, stretching, and slipping movement.	Optimize sintering temperature	[145]
		Add a suitable binder, which prevents cracks	[16]
		Reduce the tensile strength	[40]
		Use self-healing polymer	[174,175]
		Use the encapsulation layer of elastomer to protect patterns	[23]

**Table 3:** Printed wearable sensors, properties, and performance.

Wearable Sensor	Material	Printing Method	Sensibility	Mechanical Properties	References
Temperature Sensor	Cu−CuNi, Kapton	aerosol	0–232 °C SC^1^ = 43.68 ± 0.35 μV/ °C (after 200 cycles of bending and twisting)	Angle of twist = 120° (Degradation of SC = 2.5%)	[34]
Temperature Sensor	CNT in SEBS^2^, thin- film FET-based	Screen	11 °C–55 °C(SC = −24.2 mV/°C	Uniaxial strains ≈ 60%	[25]
Strain Sensor	MWCNT, Si polymer	inkjet	GF = 1.0 (Ratio MWCNT:silicon = 1:15)	Up to 300% (max. hysteresis = 11%)	[188]
Strain Sensor	AgNP/MWCNT nanocomposite,PDMS	aerosol	GF = 58.7 (D < 5% during 1000 times loading-unlading cycle)	Up to 74%	[83]
Strain Sensor	AgNP, PEN^3^	Inkjet	GF = 3500 RT 154 ms (>4500 cycles)	1.10%	[24]
Pressure Sensor	AgNP, PEN	Inkjet	<2.9 kPa (0.0049 kPa^−1^) and >2.9 kPa (0.081 MPa^−1^) (>5700 cycles)	-	[24]
Pressure Sensor	AgNW(electrode), PVF^4^/NM(substrate), TPU^5^/NM^6^(dielectric layer)	Screen	S = 4.2 kPa^−1^ RT < 26 ms	-	[57]
Electrochemical Sensor-glucose	GOx^7^/Pt-graphite as WE Ag/AgCl as RE Pt wire as CE PU^8^ as substrate	Screen	33 μM–0.9 mM	Up to 75%	[189]
Electrochemical Sensor–lactate, cortisol	e-RGO^9^ with cortisol and lactate antibodies	Screen	0.1 ng mL^−1^ for cortisol/0.1 mM for lactate	-	[71]

^1^ SC = Seebeck coefficient. ^2^ SEBS = styrene-ethylene-butadiene-styrene. ^3^ PEN = polyethylene naphthalate. ^4^ PVF = polyvinylidene fluoride. ^5^ TPU = thermoplastic polyurethane. ^6^ NM = nanomembrane. ^7^ GOx = glucose oxidase. ^8^ PU = polyurethane. ^9^ e-RGO = electroreduced graphene oxide.
